# Hydrogen cyanamide induces grape bud endodormancy release through carbohydrate metabolism and plant hormone signaling

**DOI:** 10.1186/s12864-019-6368-8

**Published:** 2019-12-30

**Authors:** Dong Liang, Xiaojing Huang, Yanqiu Shen, Tian Shen, Huifen Zhang, Lijin Lin, Jin Wang, Qunxian Deng, Xiulan Lyu, Hui Xia

**Affiliations:** 1 College of Horticulture, Sichuan Agricultural University, Chengdu, 611130 Sichuan China; 2 Institute of Pomology and Olericulture, Sichuan Agricultural University, Chengdu, 611130 Sichuan China

**Keywords:** Grape, Endodormancy release, Hydrogen cyanamide, Carbohydrate, Plant hormone

## Abstract

**Background:**

Grape buds exhibit non-uniform, or delayed, break in early spring in subtropical regions because the accumulation of chilling is insufficient. Hydrogen cyanamide (H_2_CN_2_, HC) can partially replace chilling to effectively promote bud sprouting and is used widely in warm winter areas. However, the exact underlying mechanism of grape bud release from endodormancy induced by HC remains elusive.

**Results:**

In this study, the transcriptome of grape winter buds under in vitro conditions following HC and water treatment (control) was analyzed using RNA-seq technology. A total of 6772 differentially expressed genes (DEGs) were identified. Furthermore, the gene ontology (GO) and Kyoto Encyclopedia of Genes and Genomes (KEGG) analysis revealed that starch and sucrose metabolism and plant hormone signaling transduction were most enriched out of the 50 total pathways. HC treatment induced the upregulated expression of sucrose synthase (SUS), sucrose phosphate synthase (SPS), α-amylase (AM), and β-amylase (BM) and downregulated expression of sucrose invertase (INV), hexokinase (HK), fructokinase (FK), soluble starch synthase (SS), and granule-bound starch synthase (GBSS). Hence, the starch concentration in the HC-treated group was significantly lower than that in control, whereas soluble sugar content in the HC-treated group increased quickly and was higher than that in control between 0 and 8 d. The concentration of indoleacetic acid (IAA) and zeatin (ZT) increased, whereas that of abscisic acid (ABA) and gibberellin (GA) decreased in HC treated group, which coincided with the expression level of genes involved in above hormone signals. The content of hydrogen peroxide (H_2_O_2_) and enzyme activity of superoxide dismutase (SOD) and peroxidase (POD) were increased in grape buds with HC treatment, whereas catalase (CAT) activity was decreased. HC treatment increased the expression of *POD*, *SOD*, primary amine oxidase (*PAO*), polyamine oxidase (*PAOX*), and glutathione peroxidase (*GSH-Px*).

**Conclusion:**

Based on these results, it is possible to propose a mechanistic model that underlies the regulation of endodormancy release in grapevine buds by exogenous HC application.

## Background

Grape (*Vitis vinifera* L.) is an important deciduous fruit tree worldwide and table grapes are the main horticultural crop in China. According to data from the United Nations Food and Agriculture Organization (FAO), in the past decade, China’s grape cultivation area increased by more than 336,919 ha and reached 778,585 ha in 2017 and the yield reached 13,160,811 tons. China is the biggest contributor to the world’s table grape industry [1]. Currently, the grape planting area has expanded from the eastern and northern regions to the western and southern regions in China. Provinces in these regions have become important areas for China’s grape industry, such as Sichuan, Yunnan, Zhejiang, and Guangxi [2]. However, these regions climate is a mid-subtropical, damp-heat eco-type zone with a warm winter [2]; thus, effective chilling accumulation is insufficient in these areas, which leads to non-uniform, or delayed, bud break in early spring. This is a major obstacle for the commercial production of table grapes in the above regions.

A certain amount of chilling accumulation is required in perennial species for the proclamation of the buds from endodormancy. Insufficient cold accumulation during chilling accumulation generally results in non-uniform flowering and a reduced fruit set [3]. In agronomic practices, some chemical reagents are often used instead of low temperature to complete natural dormancy. Hydrogen cyanamide (H_2_CN_2_, HC) is an effective chemical agent for releasing dormancy and has been successfully applied to commercial crops such as grape (*Vitis vinifera* L.) [4], apple (*Malus domestica* Borkh.) [5], sweet cherry (*Prunus avium* L.) [6], and kiwifruit (*Actinidia deliciosa*) [7]. There is evidence that HC has various influences on dormancy release in grape. HC treatment causes an abrupt increase in starch hydrolysis and the transient accumulation of soluble sugars in the bud coincides with a transient induction of α-amylase activity [8]. Exogenous HC application increases the expression level of sucrose synthase, pyruvate decarboxylase, a sucrose non-fermenting (SNF)-like protein kinase, grape dormancy breaking-related protein kinase, and alcohol dehydrogenase [9, 10]. Abscisic acid (ABA) also inhibits dormancy release in grape buds and attenuates the advancing effect of HC [11]. HC can promote dormancy release by increasing the content of ethylene and cytokinin [10, 12]. Differentially expressed genes and proteins involved in metabolic, ribosomal, and hormonal signaling pathways are significantly enriched between HC treatment and a control treatment [13]. In addition, HC application stimulates the temporary elevation in H_2_O_2_ levels and rapidly upregulates certain genes associated with oxidative stress [4, 9, 10], which causes a sharp decrease in catalase (CAT) activity and transient stimulation of peroxidase (POD) and ascorbate peroxidase (APX) activities [14]. Moreover, some reports have illustrated that HC-induced bud break is enhanced by calcium (Ca^2+^) signaling and stimulates changes in phosphorylation and transcription regulators [15].

Despite the extensive global use of HC by growers for breaking dormancy in grape, the underlying mechanism that explains the role of HC in dormancy breaking is still not clear. The aim of this study was to investigate the underlying molecular processes behind grape bud sprouting using the application of HC. In this study, RNA-seq was used to categorize and characterize the differentially expressed genes (DEGs) and pivotal pathways in different grape bud dormancy release stages. In addition, the content of products and expression of genes involved in candidate pathways was determined. This study will help us understand the mechanisms in HC-induced grape bud sprouting in regions with warm winters.

## Results

### Bud break rate

**Fig. 1:**
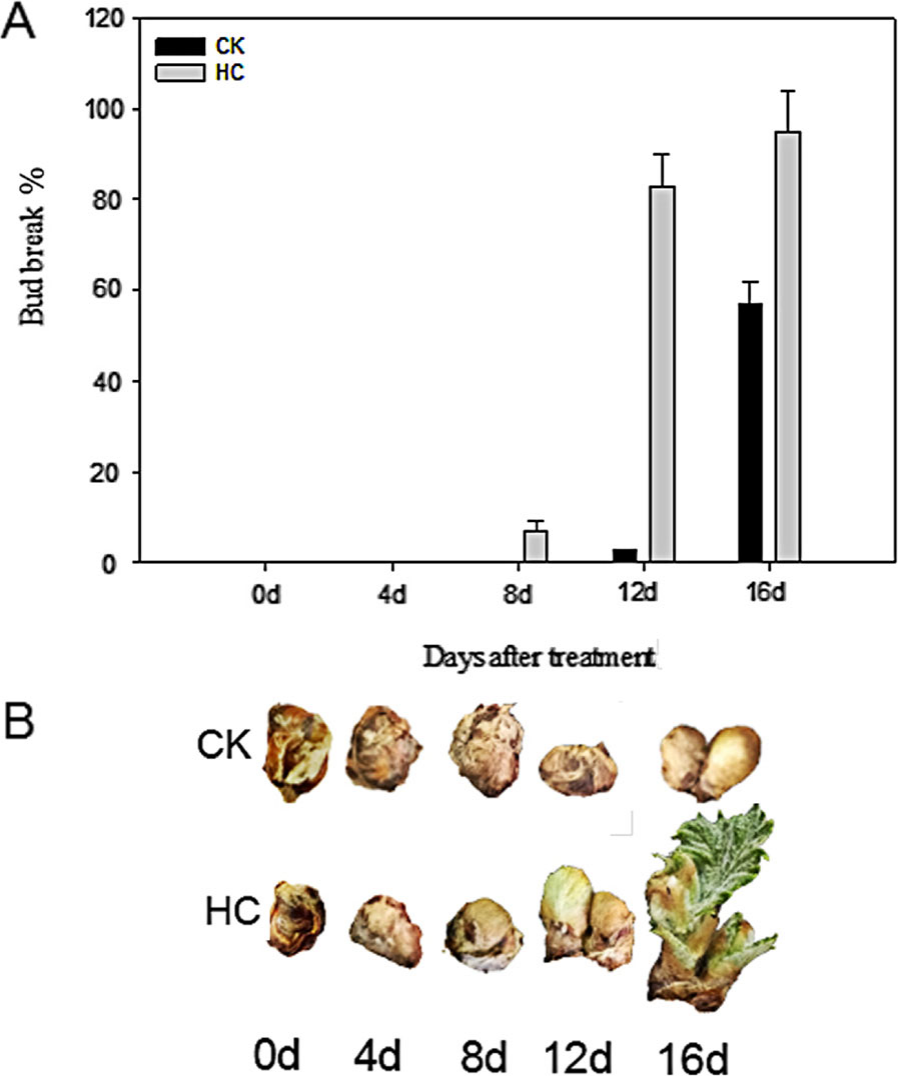
Bud breaks response of HC- and water-treated grape buds at 0, 4, 8, 12 and 16 days after treatment (**a**) Bars represent ± SEM of three biological replicates. **b** Developmental stages of the grape buds sampled in this study

To achieve controlled endodormancy release, dormant grape buds were treated with HC and the percentage of bud break was recorded. This is shown in Fig. 1a. There were no significant differences between the HC-treated and control (CK) group for the first 4 d (Fig. 1b). Sprouting was observed 8 d and 12 d after incubation by HC and water, respectively. On 16 d, the bud breaking rate was 97.85 and 57.62% in the HC treatment group and CK group, respectively. HC effectively promoted dormant release and brought sprouting time forward.

### Analysis of transcriptional sequence data

RNA sequencing analysis was used to explore how HC could break grape bud dormancy. For RNA-seq, three biological replicates from each of the following time points were selected: buds of 0 d, 4–12 d mixed sample with water treatment (CK4–12 d), and HC treatment (HC4–12 d). After the elimination of low-quality reads and adaptor sequences, a total of 240.57 million clean reads were recorded and mapped to the grape genome. Finally, of the high-quality reads generated from the three samples, uniquely mapped reads were 65.91–71.52%, whereas total mapped reads were 67.04–72.56% (Table 1).

**Table 1:** Reads number based on RNA-Seq data in three stages of grape buds

Type	0d	CK4–12d	HC4–12d
Total raw reads	156,511,930	149,786,486	174,844,788
Total mapped reads (%)	104,929,087 (67.04)	101,751,322 (68.01)	126,829,381 (72.56)
Unique mapped reads (%)	103,171,028 (65.91)	100,172,525 (66.96)	125,037,588 (71.52)
Multiple mapped reads (%)	1,758,059 (1.23)	1,578,797 (1.06)	1,809,793 (1.04)
Mean GC percentage (%)	45.59	45.83	45.82
Total clean reads	78,255,965	74,893,243	87,422,394

### Identification and functional classification of differentially expressed genes

The expression level of a gene is usually measured by FPKM (expected number of Fragments Per Kilobase of transcript sequence per Millions of base pairs sequenced). The screening criteria for a DEG was Fold Change > 4 and False Discovery Rate < 0.001. Nine samples were detected with 29,551 genes expressed. For the pairwise comparisons among the three stages, 951 (0 d vs CK4–12 d), 4047 (0 d vs HC4–12 d), and 1774 (CK4–12 d vs HC4–12 d) DEGs were detected (Table 2). It was observed that the greater abundance of DEGs in the comparison between the 0 d and HC4–12d stages were more distinct than other comparisons, which indicated that the transcriptome profiles of these two stages were much more distinct than those of the CK4–12d stages. In summary, the number of upregulated genes was greater than that of downregulated genes by pairwise comparison.

**Table 2:** Number of differentially expressed genes identified by comparing the gene expression levels between two samples

DEG Set	DEG Number	Up-regulated	Down-regulated
0d vs CK4–12d	951	783	168
0d vs HC4–12d	4047	2695	1352
CK4–12d vs HC4–12d	1774	1090	684

**Fig. 2:**
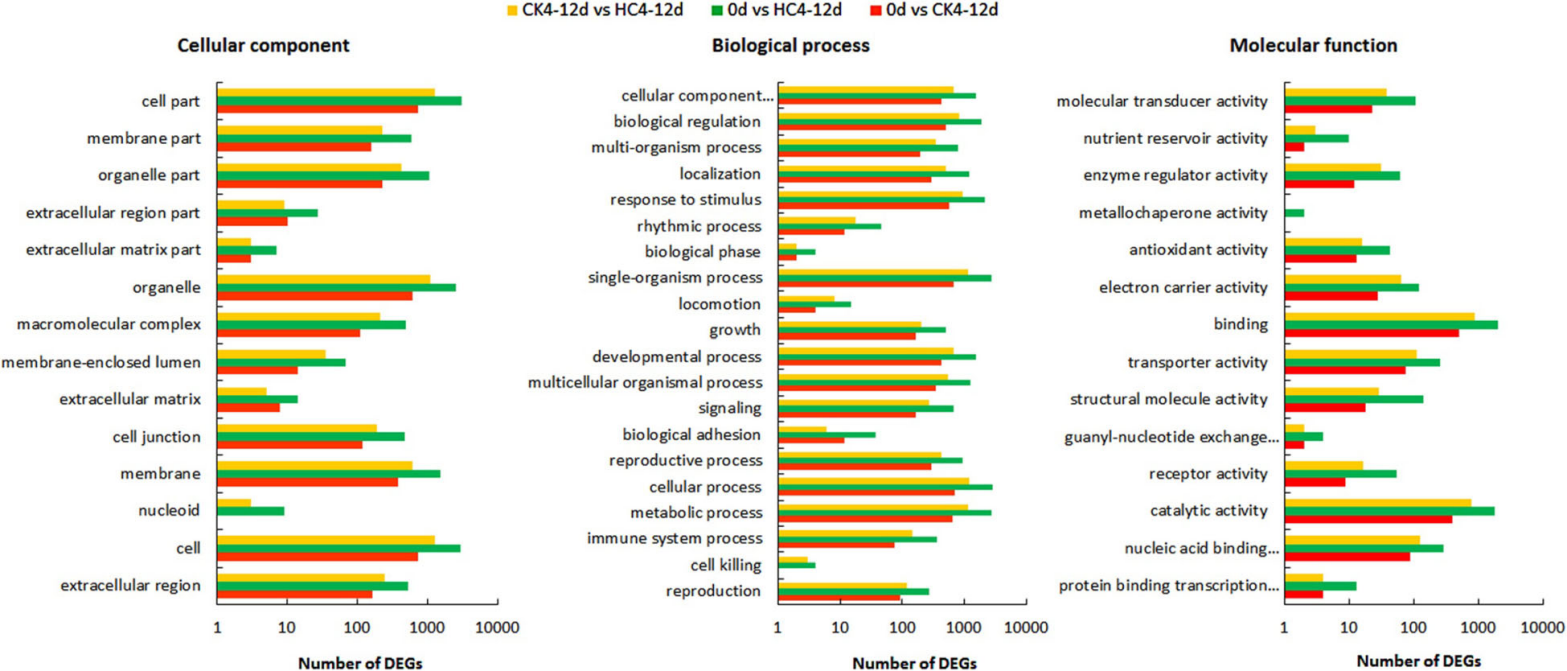
GO distributions of the transcripts differentially expressed among three dormancy stages. The transcripts were annotated into three main categories. A cellular component, B biological process and C molecular function

For gene ontology (GO), annotated genes were mainly divided into three ontologies: molecular functions (MF), biological processes (BP), and cellular components (CC). Because some genes have multiple functions, the total number of GO clustered genes will be greater than the total number of RNA-seq. The BP category contained the majority of GO annotations (222,191; 62.78%) followed by the CC category (67,034, 18.94%) and MF category (64,682, 18.28%). The major subcategories, along with the analysis of transcripts among the buds in three different stages, are shown in Fig. 2. In the transcripts annotated in GO major categories, the 0d vs CK4–12d, 0d vs HC4–12d, and CK4–12d vs HC4–12d comparisons represent 10,116, 40,080, and 16,997 transcripts, respectively.

The DEGs between each pair of stages were enriched in genes related to distinct Kyoto Encyclopedia of Genes and Genomes (KEGG) pathways. The top 15 KEGG pathways corresponding to the most abundant DEGs are presented in Additional file 1: Figure S1. The shared KEGG pathways for the 0 d vs CK4–12 d, 0d vs HC4–12 d, and CK4–12 d vs HC4–12 d comparisons included plant hormone signal transduction, starch and sucrose metabolism, protein processing in the endoplasmic reticulum, phenylpropanoid biosynthesis, pentose and glucuronate interconversions, photosynthesis, photosynthesis-antenna proteins, and biosynthesis of amino acids. Based on KEGG analysis, photosynthesis (56 transcripts), starch and sucrose metabolism (113 transcripts), and plant hormone signal transduction (91 transcripts) were the enriched pathways. Therefore, starch and sucrose metabolism and plant hormone signal transduction were mainly studied regarding HC breaking dormancy.

### Starch and sucrose metabolism

**Fig. 3:**
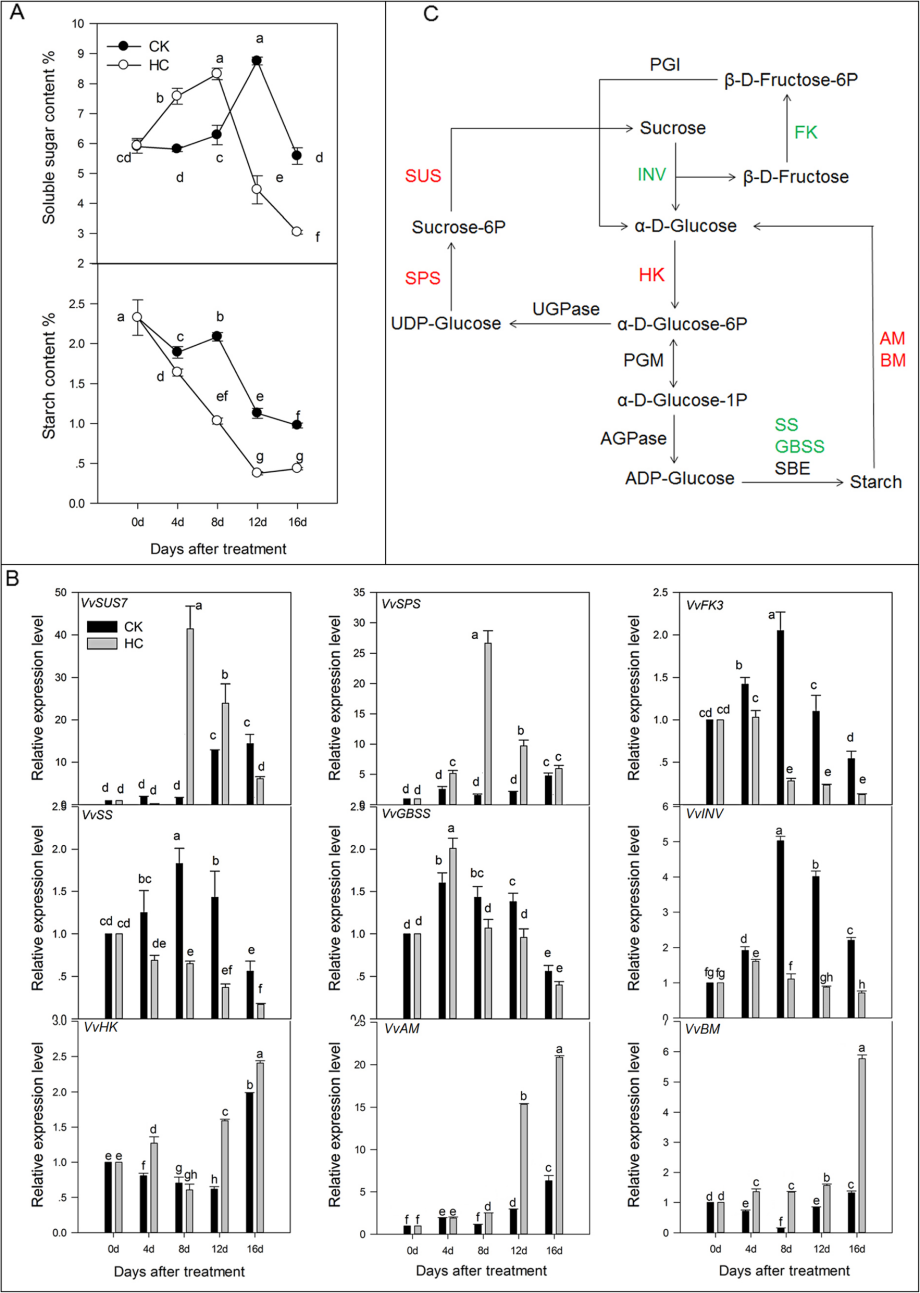
Effect of HC on starch and sucrose metabolism. Bars indicate ± SEM of three biological replicates and different letters indicate significant difference. Different letters in the figure indicate significant difference (*P* < 0.05) Red color: up-regulation; Green color: down-regulation. **a** Concentration of soluble sugar and starch in two different treatments. **b** Transcripts related to sucrose and starch metabolism analyzed by qRT-PCR. **c** Different factors in starch and sucrose metabolism pathway affected by HC treatment. AM, α-amylase; AGPase, ADP- glucose pyrophosphorylase; BM, β-amylase; FK, fructokinase; GBSS, granule-bound starch synthase; HK, hexokinase; INV, sucrose invertase; PGI, phosphoglucose isomerase; PGM, phosphoglucomutase; SBE, starch-branching enzyme; SPS, sucrose phosphate synthase; SUS, sucrose synthase; UGPase, UDP-glucose pyrophosphorylase

Soluble sugar plays an important role in plant growth and development. It provides energy for plant growth and acts as a signal molecule to regulate plant growth. In this study, soluble sugar and starch contents were determined and the expression of important DEGs involved in starch and sucrose metabolic pathways were detected by qRT-PCR. After HC treatment, the soluble sugar content peaked at 8 d and then decreased sharply. The starch content continued to decrease from 0 to 16 d (Fig. 3a). During CK sprouting, the content of soluble sugar increased from 0 to 12 d and then declined. The starch concentration in the CK was always higher than that in HC treatment. In the starch and sucrose metabolic pathways, soluble starch synthase (SS), granule-bound starch synthase (GBSS), α-amylase (AM), and β-amylase (BM) were important enzymes for starch synthesis and degradation. In HC treatment, the expression level of *VvSS* and *VvGBSS* was downregulated and that of *VvAM* and *VvBM* were upregulated at 8–16 d (Fig. 3b). Sucrose invertase (INV), hexokinase (HK), sucrose synthase (SUS), and sucrose phosphate synthase (SPS) play important roles in sucrose catabolism. The expression of *VvHK*, *VvSPS*, and *VvSUS* were significantly upregulated after HC treatment, especially at 8–16 d and was higher than that in the CK group. Nevertheless, the expression of *VvINV and VvFK3* was downregulated by HC treatment and was lower than that in the CK (Fig. 3b). Figure 3c summarizes the different factors affected by HC treatment in the starch and sucrose metabolism pathways.

### Indoleacetic acid (IAA) signal transduction

**Fig. 4:**
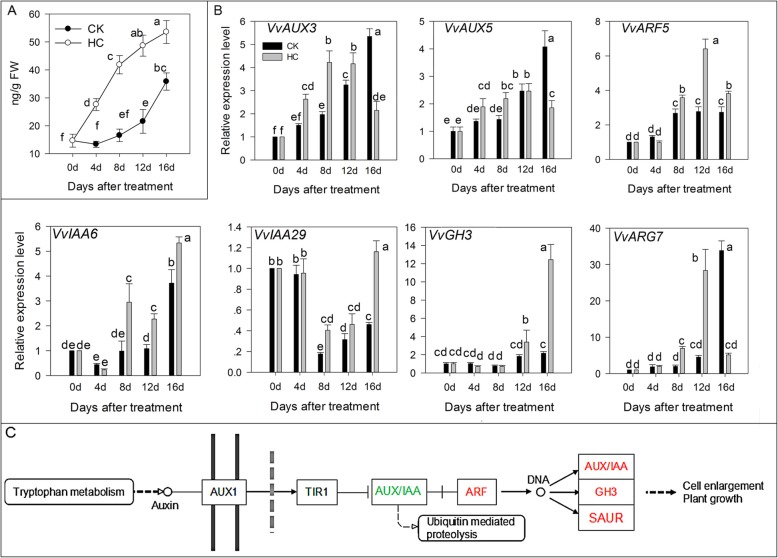
Effect of HC on IAA signals transduction. Bars indicate ± SEM of three biological replicates and different letters indicate significant difference. Different letters in the figure indicate significant difference (*P* < 0.05). Red color: up-regulation; Green color: down-regulation. **a** Concentration of IAA in two different treatments. **b** Transcripts related to IAA signal transduction analyzed by qRT-PCR. **c** Different factors in IAA signal transduction pathway affected by HC treatment. ARF, auxin response factors; AUX1, auxin / indoleacetic acids proteins; GH3, gretchen hagen 3; SAUR, small auxin-up RNA

In the IAA signal transduction pathway, auxin/indoleacetic acids proteins-auxin response factors (AUX/IAA-ARF) dimers disintegrate when IAA is present and AUX/IAA is degraded by ubiquitination to release ARFs. ARFs form homologous and heterologous dimers after transcriptional activation to regulate the expression of downstream genes and cause the auxin-related response. In the bud breaking dormancy process, IAA was accumulated by HC treatment (Fig. 4a). Although the IAA concentration was also increased in the CK group, it was significantly lower in the CK group than that with HC treatment. In the IAA signal transduction pathway, HC treatment induced *VvAUX3* and *VvAUX5* downregulation at 12–16 d. *VvARF5*, *VvIAA6*, *VvIAA29*, *VvGH3*, and *VvARG7* are response factors that directly act on plant growth in the IAA signal pathway. We observed that HC treatment induced the upregulation of these genes (Fig. 4b). Figure 4c summarizes the different factors affected by HC treatment in the IAA transduction pathway.

### Cytoplasmic protein-tyrosine kinase (CTK) signal transduction

**Fig. 5:**
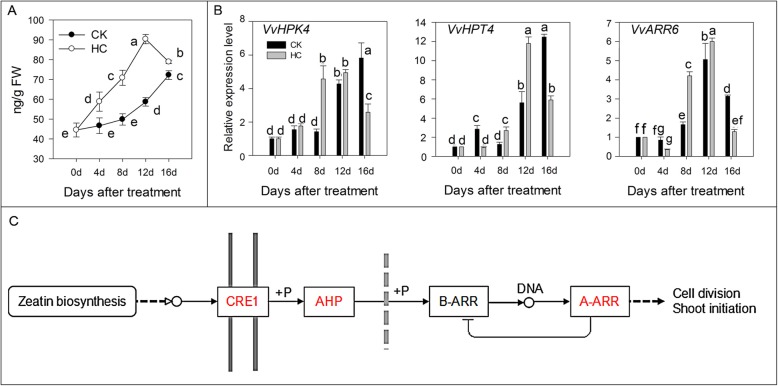
Effect of HC on CTK signals transduction. Bars indicate ± SEM of three biological replicates and different letters indicate significant difference. Different letters in the figure indicate significant difference (*P* < 0.05). Red color: up-regulation; Green color: down-regulation. **a.** Concentration of CTK in two different treatments. **b.** Transcripts related to CTK signal transduction analyzed by qRT-PCR. **c.** Different factors in CTK signal transduction pathway affected by HC treatment. ARR6, two-component response regulator 6; HK4, histidine kinase 4; HPT4, histidine-containing phosphotransfer protein 4

CTKs promote dormancy release. We observed that the zeatin (ZT) concentration increased significantly with HC treatment. At 12 d, the ZT concentration reached its peak (90.4 ng/g) in the HC-treated group and this was 1.5 times higher than that in the CK group. The ZT concentration decreased at 16 d (Fig. 5a). In the CTK signal transduction pathway, a cytokinin receptor (CRE1) binds to CTK and is self-phosphorylated. This signal is transferred to a phosphate transporter (AHP). The phosphorylated AHP enters the nucleus and transfers the phosphate group to a series of response regulators (ARR), thereby regulating the downstream cytokinin reaction and plant growth and development. Compared with the expression in the CK group, HC treatment induced *VvHPK4*, *VvHPT4*, and *VvARR6* upregulation at the sprouting stage (8–12 d) (Fig. 5b). Figure 5c summarizes the different factors affected by HC treatment in the CTK signal transduction pathway.

### Gibberellin (GA) signal transduction

**Fig. 6:**
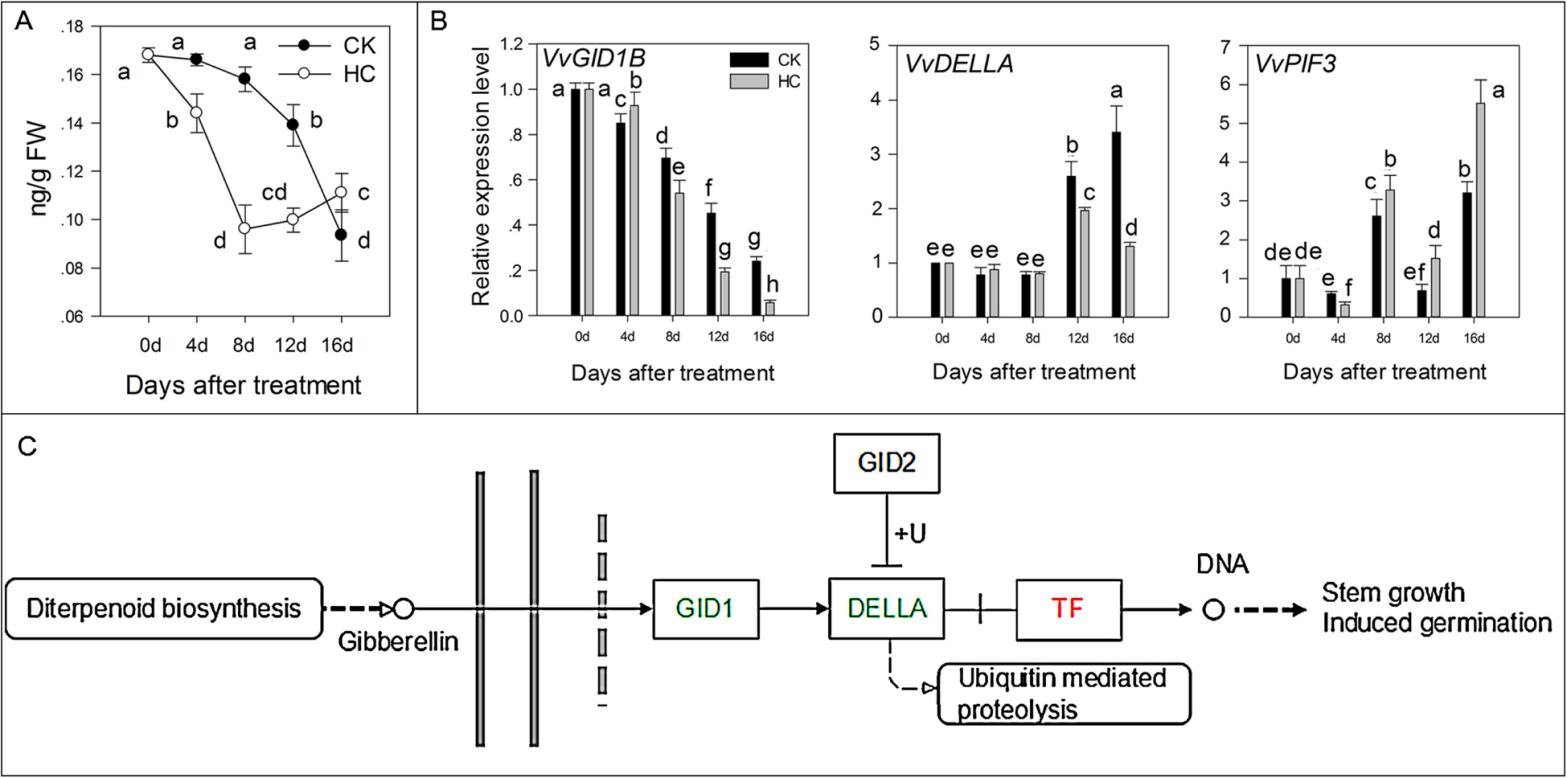
Effect of HC on GA signals transduction. Bars indicate ± SEM of three biological replicates and different letters indicate significant difference. Different letters in the figure indicate significant difference (*P* < 0.05). Red color: up-regulation; Green color: down-regulation. **a.** Concentration of GA3 in two different treatments. **b.** Transcripts related to GA signal transduction analyzed by qRT-PCR. **c.** Different factors in GA signal transduction pathway affected by HC treatment. DELLA, DELLA protein; GID1, GA-insensitive dwarf1; PIF3: phytochrome interacting factors 3; TF, Transcription factor

GA is a hormone that is indispensable for breaking seed dormancy. In the HC-treated group, GA_1_ content decreased sharply and was lowest at 8 d, then increased gradually until buds spouted, whereas GA_1_ in the CK group was stable up to 8 d and decreased markedly to 16 d (Fig. 6a). GA_1_ content in the HC treatment group was significantly lower than that in the CK group, in almost the whole process of HC-induced dormancy release. In the GA signal transduction pathway, DELLA protein inhibits the growth and development of plants in the absence of GA. When a GA signal is received by the GA signal sensing region on the DELLA protein, receptor GA-insensitive dwarf 1 (GID1) binds to DELLA to promote the release of the repression function of the DELLA protein and the plant shows normal GA reaction and growth. In this study, HC treatment downregulated the expression of *VvGID1B* and *VvDELLA*, but upregulated the expression of *VvPIF3*, which induced the expression of downstream genes related to GA anabolism to promote bud breaking (Fig. 6b). Figure 6c summarizes the different factors affected by HC treatment in the GA signal transduction pathway.

### ABA signal transduction

**Fig. 7:**
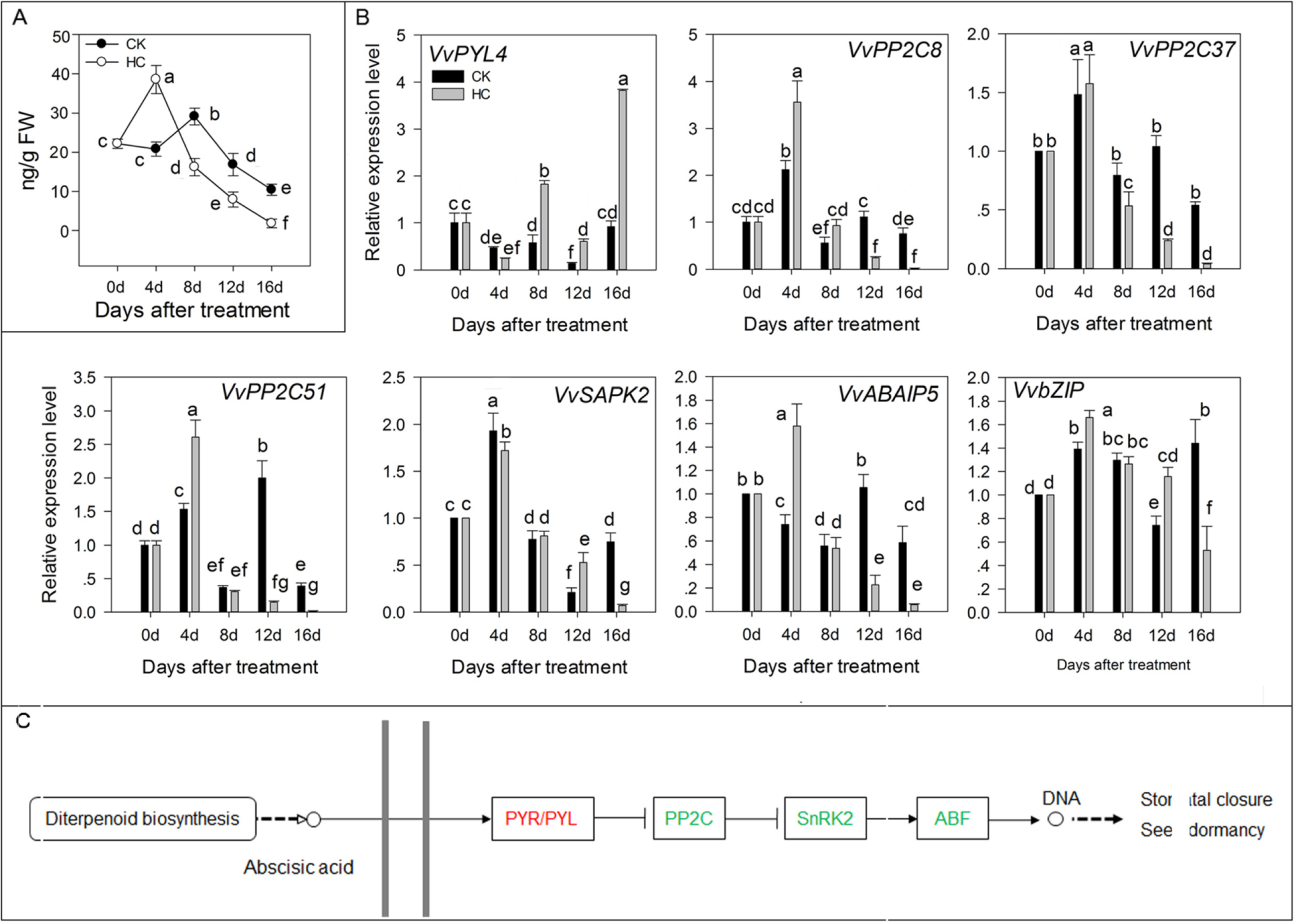
Effect of HC on ABA signals transduction. Bars indicate ± SEM of three biological replicates and different letters indicate significant difference. Different letters in the figure indicate significant difference (*P* < 0.05). Red color: up-regulation; Green color: down-regulation. **a.** Concentration of ABA in two different treatments. **b**. Transcripts related to ABA signal transduction analyzed by qRT-PCR. **c.** Different factors in GA signal transduction pathway affected by HC treatment. ABAIP, abscisic acid-insensitive 5-like protein; ABF, ABRE binding factors; bZIP, basic leucine zipper transcription factors; PP2C, protein phosphatase 2C; PYR/PYL, abscisic acid receptor; SAPK2, serine/threonine-protein kinase 2; SnRK2, SNF1-related kinase 2

ABA is closely related to plant dormancy. In the ABA signal transduction pathway, ABA combined with ABA receptor (PYR/PYL) can inhibit the enzyme activity of protein phosphatase 2C (PP2C). Serine/thread-protein kinase 2 (SNPK2) activates its own activity via autophosphorylation and acidifies different downstream targets, which triggers ABA-induced physiological reactions. In this study, the concentration of ABA increased at 4 d and then sharp decreased (Fig. 7a). ABA content began to decline after 8 d in the CK group. However, the content of ABA in the HC group decreased from 38.56 ng/g (4 d) to 1.80 ng/g (16 d) and was consistently lower than that in the CK group. In the ABA signal transduction pathway, ABA receptor *VvPYL4* was upregulated and *VvPP2C8*, *VvPP2C37*, and *VvPP2C51* were downregulated by HC treatment. Subsequently, *VvSAPK2* was activated by autophosphorylation and downregulated by HC, which induced the decreased expression of *VvABAIP5* and *VvbZIP* (Fig. 7b). Figure 7c summarizes the different factors affected by HC treatment in the ABA signal transduction pathway.

### H_2_O_2_ accumulation and antioxidative enzyme assay

**Fig. 8:**
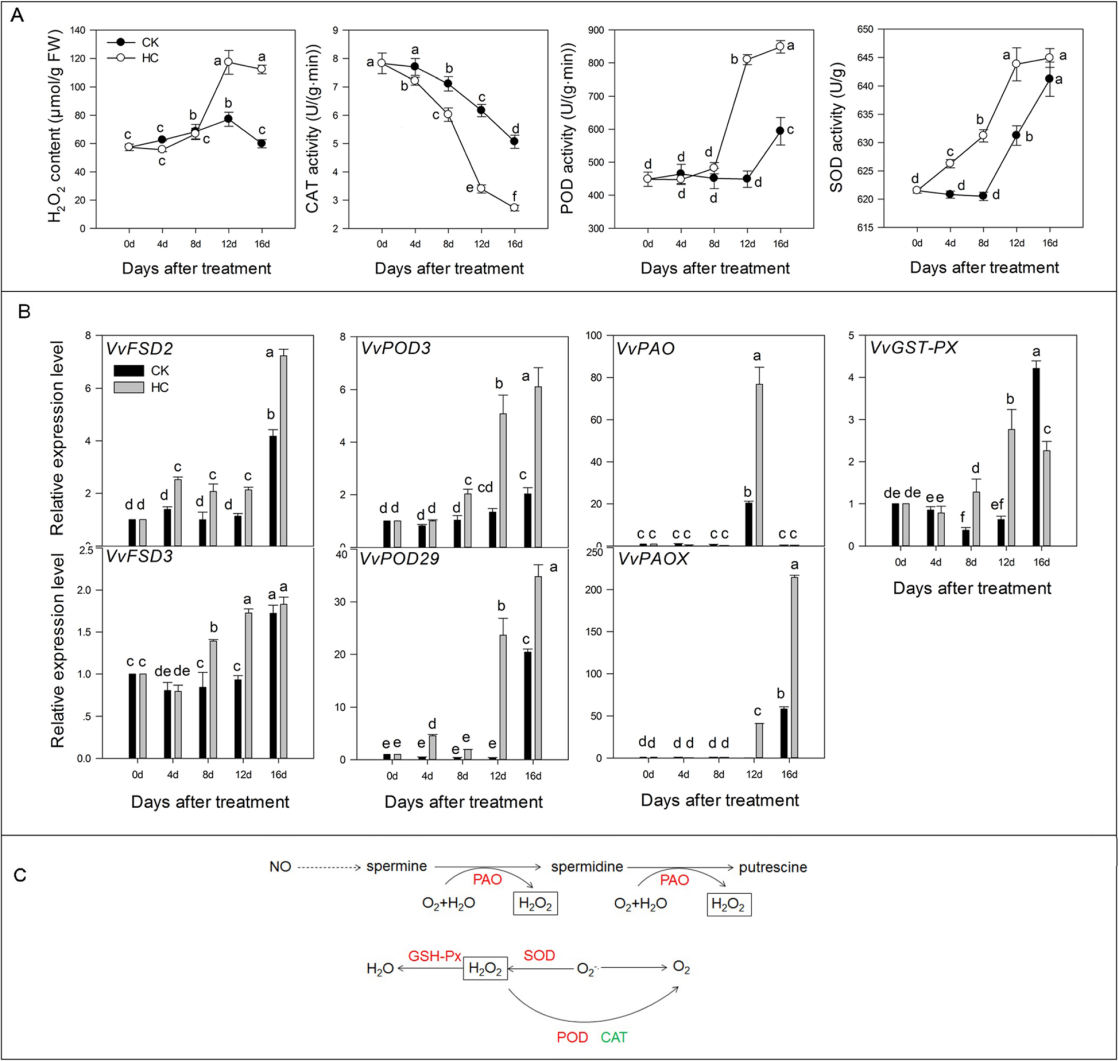
Effect of HC on oxidation system. Bars indicate ± SEM of three biological replicates and different letters indicate significant difference. Different letters in the figure indicate significant difference (*P* < 0.05). Red color: up-regulation; Green color: down-regulation. **a**. The changes of H_2_O_2_ Concentration and CAT, POD and SOD activity in two different treatments. **b**. Transcripts related to oxidation system analyzed by qRT-PCR. **c**. Different factors in oxidation system pathway affected by HC treatment. CAT, catalase; GSH-Px, glutathione peroxidase; FSD, superoxide dismutase [Fe]; PAO, primary amine oxidase; PAOX, polyamine oxidase; POD, peroxidase; SOD, superoxide dismutase

The accumulation of H_2_O_2_ in grapevine buds was determined in this study. For all treatments, H_2_O_2_ increased rapidly, reaching its maximum level after 12 d and decreased slightly thereafter (Fig. 8a). From 0 d to 8 d, there was no significant difference in H_2_O_2_ content between HC and CK. However, the averaged amount of H_2_O_2_ in the HC treated group was higher than that in control from 8 d to 16 d. CAT activity decreased sharply in the two groups with the prolongation of treatment time, but the value in the HC-treated group was lower than that in the CK group from 4 d to 16 d (Fig. 8a). When the buds sprouted, POD and SOD activity increased gradually in all treatments, reaching the peak at 16 d (Fig. 8a). There were no significant changes in POD activity between the two treatments at 0–8 d, but the POD activity in the HC-treated group was higher than that in the CK group at 8–16 d (Fig. 8a).

In the process of reactive oxygen species (ROS) metabolism, the HC-treated group induced the upregulation of ROS-generating genes (*VvFSD2*, *VvFSD3*, *VvPAO*, and *VvPAOX*) and led to the H_2_O_2_ accumulation. To maintain normal growth, the antioxidant defense system of the plant plays a role: ROS-scavenging gene (*VvPOD3*, *VvPOD29*, and *VvGSH-Px*) expression was upregulated by HC treatment (Fig. 8b). Figure 8c summarizes the different factors affected by HC treatment in the ROS metabolism pathway.

## Discussion

Carbohydrate metabolism plays an important role in regulating dormancy release processes in plants. A ratio of hexoses to starch content (10:1) appears to be associated with the end of endodormancy in sweet cherry (*Prunus avium* L.) [16]. During endodormancy, a decrease in the shoot starch concentration and an increase of soluble sugars in both the flower buds and shoots are observed in the Japanese pear (*Pyrus pyrifolia* Nakai) [17]. HC treatment also causes an abrupt increase in starch hydrolysis and a transient accumulation of soluble sugars in the bud and the internode tissues of superior seedless grapevines during the first 5 days following HC treatment [8]. In this study, the starch content declined remarkably at 0–12 d and this was followed by a slight increase with HC treatment, whereas soluble sugar content increased significantly at 0–8 d followed by a remarkable decrease from 8 to 16 d (Fig. 3a). These results indicated that the increase in soluble sugar (for example, sucrose in grape) and decrease in starch content played key roles in the dormancy release process and can be impacted by HC application. The results of this study indicated that HC treatment upregulated the expression of *VvSUS* and *VvSPS* and downregulated the expression of *VvSS* and *VvGBSS* (Fig. 3b). HC treatment also upregulated the expression of *VvAM* and *VvBM* related to starch degradation (Fig. 3b). In addition, the expression of *VvINV* was downregulated at 4–16 d after HC treatment and this downregulation was lower than that after CK treatment (Fig. 3b). Hence, the starch content declined at 0–12 d and this was followed by a slight increase with HC treatment. The trend in soluble sugar content was the opposite of this trend in starch content (Fig. 3a). We speculate that the decrease of starch along with the accumulation of soluble sugars during the bud dormancy release stage, which is attributed to the hydrolyzation of starch to soluble sugar, provides energy for the plant to resist low temperatures in winter and maintain normal growth [18]. When dormancy was relieved completely, the starch content did not change significantly and the soluble sugar content decreased significantly. This was because the plant needed a lot of energy to resume growth and soluble sugar was utilized [19].

Endogenous hormones play key roles in the promotion and inhibition of growth in a dynamic balance. A low concentration of IAA promotes bud breaking and a high concentration of IAA has no significant effect on sprouting [20]. In this experiment, although the content of endogenous IAA increased after HC treatment (Fig. 4a), it was still in the low concentration range that can promote grape bud breaking. After HC treatment, the level of trans-zeatin riboside increased sharply during grape sprouting [21]. HC breaks the dormancy of sweet cherry flower buds by increasing trans-zeatin riboside and dihydrozeatin content [6]. Hence, in this experiment, the content of ZT in the HC-treated group was significantly higher than that in the CK group (Fig. 5a).

In many cases, the determination of dormancy or sprouting mainly depends on the balance between ABA and GA levels. HC treatment leads to a decrease in the endogenous ABA level by promoting ABA degradation and inhibiting ABA synthesis, as shown in grape [11] and sweet cherry [6]. In this study, ABA concentration decreased gradually with bud breaking in the HC treatment group (Fig. 7a). This can be explained by HC treatment downregulating the expression of the key genes (*VvNCED* and *VvXERICO*) involved in ABA biosynthesis and upregulating the expression of the gene (*VvA8H3*) that encodes the ABA catabolic enzyme [22, 23]. In addition, HC treatment produced respiratory stress, which led to anaerobic respiration in cells and increased H_2_O_2_ content (Fig. 8). Studies have shown that ABA 8′-hydroxylase (ABA8’OH) mediates H_2_O_2_ and NO to break dormancy and catalyzes the degradation of ABA [24, 25]. GA has this same antagonistic relationship with ABA [26]. HC treatment causes GA biosynthesis and the degradation genes of grape to increase and decrease, respectively [27]. GA displays a rapid increase when sweet cherry buds enter the burst stage and is associated with the promotion of budburst and blooming by HC treatment [28]. Recent studies have shown that the expression of the GA biosynthesis genes *VvGA3ox2, VvGA20ox3, and VvGA20ox6* are significantly downregulated after HC treatment for 24 h and the expression of *VvGA3ox2* and *VvGA20ox6* are upregulated after HC treatment for 96 h [26]. In this study, GA content decreased at 0–8 d and then increased, but this change was not significant (Fig. 6a). It has been proposed that during the initial activation of the dormant bud meristem, the level of GA must be restricted, but after meristem activation, an increase in its level enhances primordial regrowth [26].

Plant growth is regulated not only by endogenous hormone content, but also by signaling transduction pathways. During chilling accumulation and subsequent germination sprouting of white spruce (*Picea glauca*) seeds, seed dormancy and germination sprouting may be partly mediated by the changing hormone concentrations and modulation of the interactions among central auxin-signaling pathway components (TIR1/AFB, Aux/IAA, and ARF4) [29]. The DELLA protein is an important negative regulator of the GA reaction, but it has a positive correlation with ABA [30]. ABA level is positively regulated by the DELLA protein by upregulating the expression of the *XERICO* gene [31]. In this study, we observed that HC treatment resulted in the upregulation (*AUX/IAA*, *ARF*, and *CYCD*) and downregulation (*DELLA* and *ABF*) of key genes. These genes are associated with hormone signaling pathways during grape bud breaking. These results indicated that the phytohormone signaling transduction pathway plays key roles in dormancy release in response to HC; however, the accurate regulatory mechanism is not clear even now.

In previous studies of accumulation, some researchers have linked a decrease in starch content during dormancy release to hormones. ABA is a hormone that promotes plant dormancy. Studies have shown that ABA inhibits AA activity during dormancy [32]. Rubio et al. [33] suggested that ABA content was positively correlated with starch content during dormancy in grape buds. In our study, ABA-induced the expression of *VvSS1* and *VvSS3* and inhibited the expression of *VvSUSs*, *VvSPS*, and *VvINVs*. The results of this experiment also suggested that HC treatment caused the concentration of ABA to decrease (Fig. 7a), which led to an increase in the activity of AA and promoted starch catabolism and soluble sugar accumulation (Fig. 3a). However, more studies are needed to explore how phytohormones induces gene expression and enzyme activity.

Many experiments have shown that H_2_O_2_ content increases in buds with HC treatment [4, 6, 11], which is the major factor responsible for bud break. H_2_O_2_ treatment inhibits CAT activity, which then generates respiratory stress, leading to cell hypoxia and anaerobic respiration [14]. On one hand, H_2_O_2_ is used as a signal molecule in cell wall loosening and leads to buds sprouting [4, 6]. On the other hand, anaerobic respiration produces ethylene, which activates related transcription factors to promote cell elongation and formation, then bud dormancy release [9, 10]. In our study, HC induced an increase in H_2_O_2_ content and a decrease in CAT activity than that in the CK group from 8 to 16 d; therefore, to scavenge redundant ROS, the activity of SOD and POD were upregulated, along with key genes involved in protective enzyme metabolism, such as *FSD*, *POD*, *PAO*, *GST-Px*, and *PAOX* (Fig. 8). The same conclusion was reached with Seedless grapevine buds [14].

## Conclusion

**Fig. 9:**
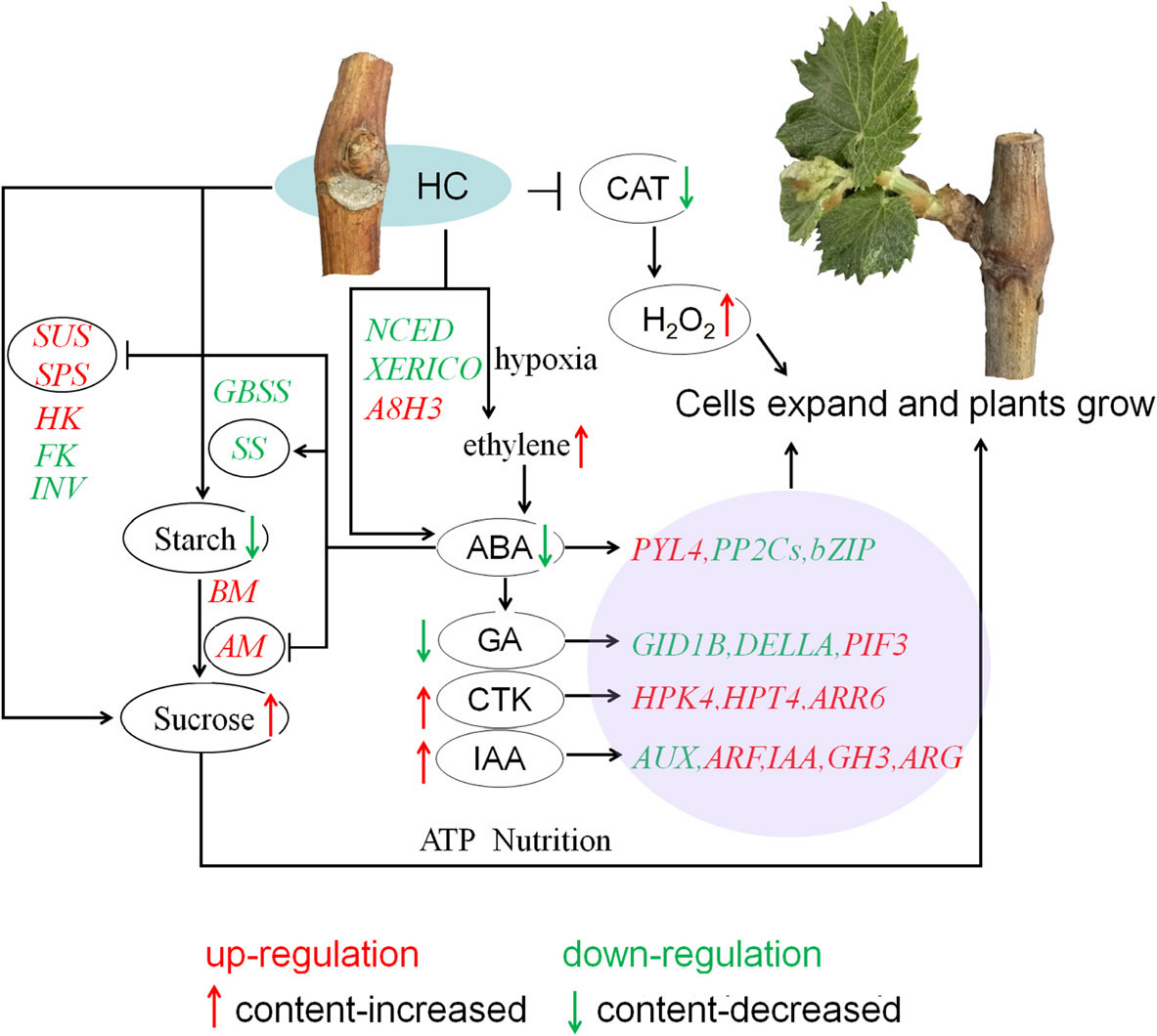
Proposed mechanistic model for dormancy release by HC in grape buds

In summary, this study provided an analysis of the metabolic changes during controlled dormancy release in grape. The results suggest a mechanism of action for HC-induced buds break that involves the activation of two pathways: sucrose and starch metabolism and plant hormone signal transduction. As shown in Fig. 9, we obtained a working model of HC breaking winter buds dormancy in grapes, which reveals the relationship between the two pathways.

## Material and methods

### Plant material

Four-year-old grapevine trees (*Vitis vinifera* L. cv. Summer black) were grown in an experimental orchard at the Sichuan Agricultural University, Chongzhou (30°24′N, 103°59′E), Sichuan province, China. The 1.5 × 3.0 m planted vines were exposed to standard horticultural practices under a rain shelter protected with polyvinyl film.

### HC treatment and sampling

At the end of December 2016 (according to production experience, grape buds were in deep dormancy during this period), the detached canes, each carrying 8 buds at positions 5–12 in the node order, were transferred to the lab. Canes were cut into single-node cuttings, randomly mixed, and separated into two groups (300 single-bud cuttings per group). Two groups were treated with 2% Dormex (520 g/L HC, AlzChem, Germany) and water (as CK). The conditions in the artificial culture room were as follows: culture temperature 25 °C, light intensity 40 μmol/m^2^∙s, day/night: 12 h/12 h, and relative humidity 80%. Sprouting was defined as the opening of bud scales to reveal new green leaves. Percent bud break was determined at 0, 4, 8, 12, and 16 d after treatment in each treatment group. HC-treated and the CK group were sampled at 0, 4, 8, 12, and 16 days, always between 3 and 5 PM. At every sampling point, buds from 60 cuttings were homogenized and divided into 6 biological replicates for different detections. Samples were frozen immediately in liquid nitrogen and kept at − 80 °C until analysis.

### Preparation of RNA-seq libraries

For RNA-seq, three biological replicates from each of the following time points were selected: buds of 0 d, 4–12 d mixed sample with water treatment and 4–12 d mixed sample with HC treatment (those equal in quality at 4 d, 8 d, and 12 d were mixed). In total, 1 μg of RNA per sample was used as input material for the RNA sample preparations. Sequencing libraries were generated using the NEBNext Ultra™ RNA Library Prep Kit for Illumina (NEB, USA) following the manufacturer’s recommendations. The library fragments were purified with an AMPure XP system (Beckman Coulter, Beverly, USA). Then, 3 μL of USER Enzyme (NEB, USA) was used with size-selected, adaptor-ligated cDNA at 37 °C for 15 min followed by 5 min at 95 °C before PCR. Then, PCR was performed with Phusion High-Fidelity DNA polymerase, Universal PCR primers, and Index (X) Primer. Finally, PCR products were purified (AMPure XP system) and library quality was assessed on the Agilent Bioanalyzer 2100 system.

### Clustering and sequencing

The clustering of the index-coded samples was performed on a cBot Cluster Generation System using TruSeq PE Cluster Kit v4-cBot-HS (Illumia) according to the manufacturer’s instructions. After cluster generation, the library preparations were sequenced on an Illumina Hiseq Xten platform and paired-end reads were generated.

### Differential expression analysis

Differential expression analysis of the two groups was performed using the DESeq R package (1.10.1). DESeq provides statistical routines for determining differential expression in digital gene expression data using a model based on the negative binomial distribution. The resulting *P* values were adjusted using the Benjamini and Hochberg’s approach for controlling the false discovery rate. Genes with an adjusted *P*-value <0.05 by DESeq were assigned as differentially expressed.

### GO and KEGG pathway enrichment analysis

GO enrichment analysis of the DEGs was implemented by the GOseq R packages based on Wallenius’ non-central hypergeometric distribution [34], which can adjust for gene length bias in DEGs. KEGG is a database resource for understanding high-level functions and utilities of a biological system, such as the cell, organism, and ecosystem. KEGG can process molecular-level information, especially large-scale molecular datasets generated by genome sequencing and other high-throughput experimental technologies (http://www.genome.jp/kegg/) [35]. KOBAS software was used to test the statistical enrichment of DEGs in KEGG pathways.

### Determination of soluble sugar and starch concentration

Anthrone colorimetry was used for the determination of soluble sugar and starch [36]. The content of soluble sugar was based on a standard curve generated with known soluble sugar concentrations. The soluble sugar standard curve was used to calculate sample sugar content. Starch content was converted into a soluble sugar content of 0.9 times. The absorbance was determined at 630 nm.

### Quantitation of plant endogenous hormone

The concentrations of four hormones, ZT, IAA, ABA, and GA, were determined by high-performance liquid chromatography (HPLC) using Agilent 1260. Three biological replicates for every sample were performed for endogenous hormone extraction in buds. Buds (0.5 g) were weighed, ground in liquid nitrogen, and subjected to ultrasonic extraction with an extraction solvent (0.5% formic acid and 80% methanol) for 30 min. After centrifugation for 15 min, the supernatant was collected. The extraction was repeated twice and the supernatant was merged. After rotary vacuum evaporation at 38 °C, the water phase was retained and centrifuged for 10 min. The supernatant was concentrated with N-EVAP and dissolved with acetonitrile. All centrifugation was performed at 12000 *g* at 4 °C. Finally, the extract was filtered with a 0.22 μm filter for HPLC detection. The chromatographic conditions were as follows: C18 (Agilent Zorbax SB) reversed phase column (250 mm × 4.6 mm), flow velocity at 0.8 mL/min, injection volume of 15 μL, and detection wavelength of 270, 218, 200, and 270 nm for ZT, IAA, ABA, and GA, respectively. The endogenous hormone contents were calculated from the peak area of each compound to internal standards (SIGMA-ALDRICH, Oakville, ON, Canada).

### Measurement of H_2_O_2_ concentration and activity of antioxidant enzymes

The determination of H_2_O_2_ content was based on the method of Lin et al. [37]. Briefly, 0.3 g of sample was ground into homogenate with 5 mL of cold acetone and centrifuged at 10000 *g* for 10 min at 4 °C. Then, 1 mL of supernatant was mixed with 0.1 mL 5% (w/v) of titanium sulfate and 0.2 mL of concentrated ammonia, centrifuged, and the supernatant was discarded. Acetone was used to wash the precipitation 3–5 times and the precipitation was dissolved with 5 mL of 2 M concentrated sulfuric acid. Finally, the volume was adjusted to 10 mL with distilled water. The absorbance was determined at 415 nm. The content of H_2_O_2_ was based on a standard curve generated with known H_2_O_2_ concentrations.

The activity of POD, SOD, and CAT were determined using the method of Liang et al. [38]. Sample (0.5 g) was ground into homogenate in 8 mL of cold 50 mM phosphate-buffered saline (PBS) (pH 7.8), containing 1% (w/v) polyvinylpyrrolidone (PVP), 0.1 mM ethylenediaminetetraacetic acid (EDTA), and 2 mM dithiothreitol (DTT). The homogenate was then centrifuged at 10000 *g* for 10 min at 4 °C. POD activity was determined by the guaiacol colorimetric method. The reaction mixtures containing 50 mM PBS (pH 5.7), guaiacol, and 30% (v/v) H_2_O_2_ were used to measure POD activity. The absorbance change in 470 nm was monitored and the result was expressed as U/g. The activity of SOD was determined by the nitro-blue tetrazolium method (NBT) and the absorbance was determined at 560 nm. The result was expressed as U/g. The reaction mixture contained 200 mM PBS (pH 7.8), 100 mM H_2_O_2_, and the enzyme extract. The activity of CAT was determined at 240 nm and the result was expressed as U/g.

### Quantitative real-time PCR

Total RNA was extracted from frozen grape buds using a modified cetyltrimethylammonium bromide method and treated with RNase-free DNase I (Takara, Dalian, China) to remove genomic DNA contamination. The forward and reverse primers for candidate and reference genes (Additional file 1: Table S1) were designed using the Primer Express® Software v2.0 (ABI, USA). Quantitative real-time PCR was performed on the CFX96 Real-Time System C1000 Thermal Cycler (Bio-RAD, Hercules, CA, USA), following the manufacturer’s protocol in an SYBR Premix Ex Taq kit (TaKaRa, Dalian, China) and analyzed with 2^−∆∆CT^. Three replicates were performed for three separate RNA extracts from three samples.

## Supplementary information

**Additional file 1: Figure S1.** Number of DEGs up and down-regulated in most enriched pathways among three stages of dormancy. **Table S1.** Primers used in this study.

## Data Availability

The datasets generated and analyzed during the current study are available in the NCBI repository [PRJNA558570]. Other supporting data can be found within the manuscript and its additional files.

## Abbreviations

ABA: Abscisic acid
ABAIP: Abscisic acid-insensitive 5-like protein
ABF: ABRE binding factors
AGPase: ADP- glucose pyrophosphorylase
AM: α-amylase
ARF: Auxin response factors
ARR6: Two-component response regulator 6
AUX1: Auxin/indoleacetic acids proteins
BM: β-amylase
bZIP: Basic leucine zipper transcription factors
CAT: Catalase
DEGs: Differentially expressed genes
DELLA: DELLA protein
FK: Fructokinase
FSD: Superoxide dismutase [Fe]
GA: Gibberellin
GBSS: Granule-bound starch synthase
GH3: Gretchen hagen 3
GID1: GA-insensitive dwarf1
GO: Gene ontology
GSH-Px: Glutathione peroxidase
H_2_O_2_: Hydrogen peroxide
HC: Hydrogen cyanamide
HK: Histidine kinase
HPT4: Histidine-containing phosphotransfer protein 4
IAA: Indoleacetic acid
INV: Sucrose invertase
KEGG: Kyoto encyclopedia of genes and genomes
PAO: Primary amine oxidase
PAOX: Polyamine oxidase
PGI: Phosphoglucose isomerase
PGM: Phosphoglucomutase
PIF3: Phytochrome interacting factors 3
POD: Peroxidase
PP2C: Protein phosphatase 2C
PYR/PYL: Abscisic acid receptor
SAPK2: Serine/threonine-protein kinase 2
SAUR: Small auxin-up RNA
SBE: Starch-branching enzyme
SnRK2: SNF1-related kinase 2
SOD: Superoxide dismutase
SPS: Sucrose phosphate synthase
SS: Starch synthase
SUS: Sucrose synthase
TF: Transcription factor
UGPase: UDP-glucose pyrophosphorylase
ZT: Zeatin
