# Supplementary material for: Hydrogen cyanamide induces grape bud endodormancy release through carbohydrate metabolism and plant hormone signaling
Source: BMC Genomics. 2019 Dec 30;20:1034. doi: 10.1186/s12864-019-6368-8 (PMC6937986; doi:10.1186/s12864-019-6368-8)
Supplement: Supplementary file 1 — Additional file 1: Figure S1. Number of DEGs up and down-regulated in most enriched pathways among three stages of dormancy. Table S1. Primers used in this study. [file 12864_2019_6368_MOESM1_ESM.doc]

Additional file 1: Figure S1


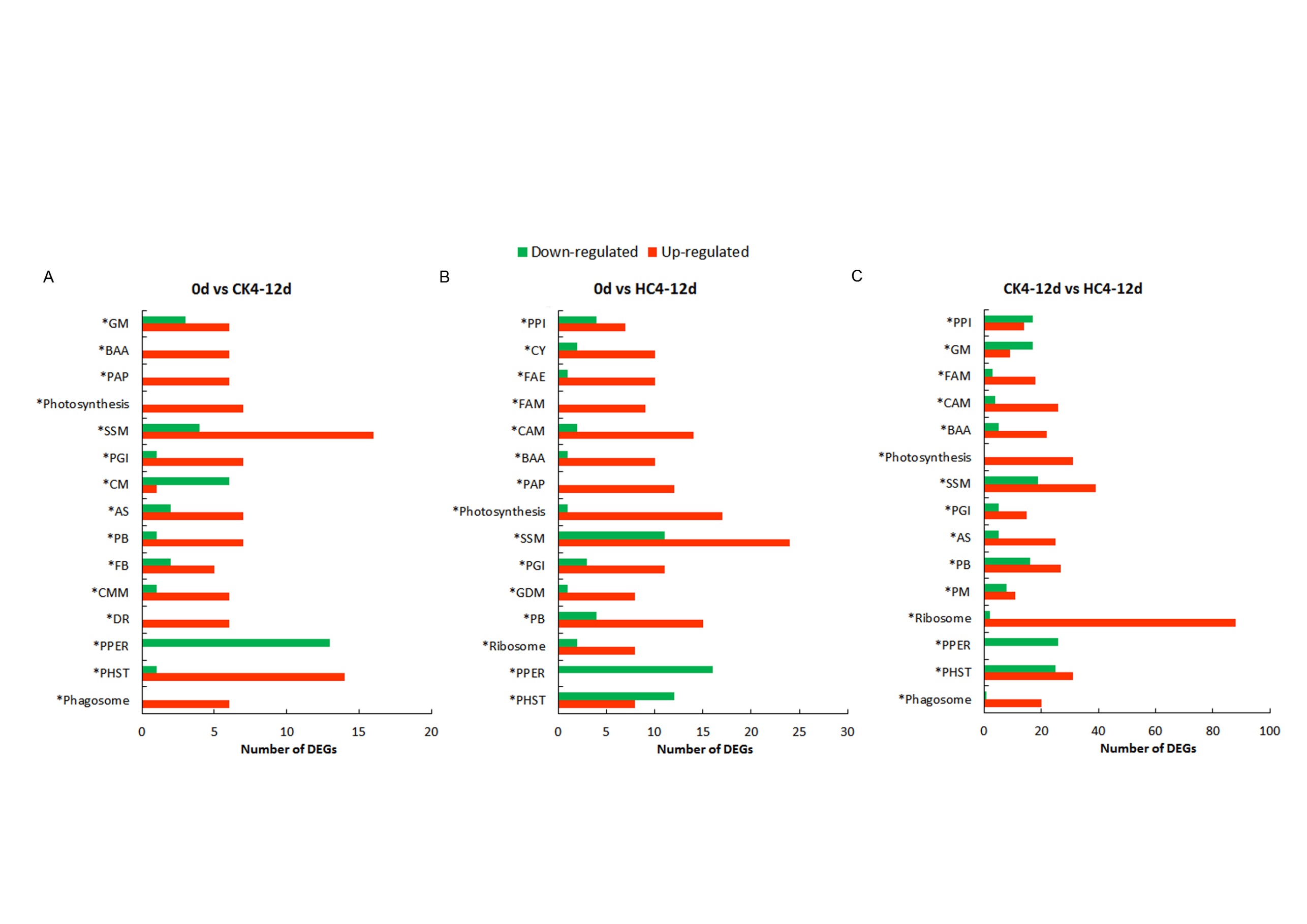


Number of DEGs up and down-regulated in most enriched pathways among three stages of dormancy. Enriched pathways were significantly enriched (*p< 0.05) during three comparative stages. A DEGs number and enriched pathways between 0d vs CK8d. B DEGs number and enriched pathways between 0d vs HC8d. C DEGs number and enriched pathways between CK8d vs HC8d. Abbreviations: AS, Amino sugar and nucleotide sugar metabolism; BAA, Biosynthesis of amino acids; CAM, Carbon metabolism ; CM, Calactose metabolism; CMM, Cysteine and methionine metabolism ; CY, Cyanoamino acid metabolism; DR, DNA replication; FAE, Fatty acid elongation; FAM, Fatty acid metabolism; FB, Flavoinoid biosynthesis; GDM, Glyoxylate and dicarboxylate metabolism; GM, Glutathione metabolism; PAP, Photosynthesis-antenna proteins; PB, Phenylpropanoid biosynthesis; PGI, Pentose ang glucuronate interconversions; PHST, Plant hormone signal transduction; PM, Phenylalanine metabolism; PPER, Protein processing in endoplasmic reticulum; PPI, Plant-pathogen interaction; SSM, Starch and sucrose metabolism.

Additional file 1: Table S1 Primers used in this study

| Gene name | Pimer | Squence | Gene ID |
| --- | --- | --- | --- |
| Grape *ACTIN* | F | GATTCTGGTGATGGTGTGAGT |  |
|  | R | GACAATTTCCCGTTCAGCAGT |  |
| *AM* | F | TAAAGGGACAGTTCTGGCGT | VIT_18s0001g00560 |
|  | R | AGCCTGTGTTGAGCCTGTAT |  |
| *BM* | F | GGTGGGGAATTGTGGAATCG | VIT_12s0059g02670 |
|  | R | CCAACATTCCCACCACACTG |  |
| *FK* | F | TGGTGAGAAGGGTTGCAGAT | VIT_15s0048g01260 |
|  | R | GAAGCCTTCCACTCTCCCTT |  |
| *GBSS* | F | GCCCAGCCCACAATGTAAAA | VIT_02s0025g02790 |
|  | R | CCTGGAGCTCTTCAAGTGGA |  |
| *HK* | F | GTCATGGGAAAAGTTGCGGT | VIT_09s0002g03390 |
|  | R | TGCACAATCCTCCTCGAAGT |  |
| *INV* | F | GCAGCAGAAATGGGGTTGAA | VIT_04s0008g01140 |
|  | R | AGGTATCGGTTTCAGGCACA |  |
| *SPS* | F | CGCAAGCCTATGATACTCGC | VIT_11s0118g00200 |
|  | R | GCAAGCTCTCTCAATGGACG |  |
| *SS* | F | TAGGTGGACGTTTTCTCCCC | VIT_16s0098g01780 |
|  | R | GGAGGAACCCAGAACCAAGA |  |
| *SUS* | F | TCCAGGGCTGTGTAGAGTTG | VIT_04s0079g00230 |
|  | R | TCGCTTGTGTCTCTCCATGT |  |
| *ARF5* | F | AGGTGAAGCTGAATGGGGAA | VIT_18s0001g13930 |
|  | R | TGGAATTGTGGGGTACGGAA |  |
| *ARG7* | F | GCAAGCTGAGGAAGAGTTCG | VIT_18s0001g13980 |
|  | R | TGGAATTGTGAGGCCCATCT |  |
| *AUX3* | F | TGGAAAGGGAAGAAGGCGAT | VIT_18s0001g03540 |
|  | R | ACCAGGCATCATAGACCGAG |  |
| *AUX5* | F | GAGACAGTGATGGTGGGGAA | VIT_03s0038g02140 |
|  | R | CATACACAGAGCCACCATGC |  |
| *GH3* | F | CGGGTTTGGCATCATGGATT | VIT_12s0059g01870 |
|  | R | GGCAAGTCCCCAGCATAATG |  |
| *IAA6* | F | ACTACCAAACCTACGCCTCC | VIT_04s0008g00220 |
|  | R | AGGATGGCAAGTTCAGAGCT |  |
| *IAA29* | F | TCCCACAACAGAGACGACAA | VIT_11s0016g05640 |
|  | R | CCACTCCCACCATCTTCACT |  |
| *ARR6* | F | GAGGAAGGTGCAGAGGAGTT | VIT_17s0000g07580 |
|  | R | CCTCTCCCTCTTCCTCTCCT |  |
| *HPK4* | F | ATTCAGCTCGACTCGTCCTT | VIT_01s0011g06190 |
|  | R | TCCCTGCATAGACCCACAAC |  |
| *HPT4* | F | ACGGCTGATCGAGAGTAAGG | VIT_09s0002g03520 |
|  | R | ACCTTTTGTAGCAGCTCCCT |  |
| *DELLA* | F | ACTCATCAGTTGACGGTGGT | VIT_11s0016g04630 |
|  | R | ATCCAGCCAAGGGTAGATCG |  |
| *GID1B* | F | TTGGTGGGCAAGAGAGAACT | VIT_07s0104g00930 |
|  | R | AAGCCCTCCAGTACCAATCC |  |
| *PIF3* | F | TCCTTTACACGCAGGGTCAT | VIT_07s0005g02510 |
|  | R | AGAACCCGGATGCTCTACTG |  |
| *ABAIP5* | F | GTGGATGAGGTGTGGAAGGA | VIT_12s0055g00420 |
|  | R | ACATCTTCCTCCCTCACTGC |  |
| *bZIP* | F | TCACTTGTGGCATGGTTTGG | VIT_18s0001g10450 |
|  | R | ATGCATTGTGGACCCAAACC |  |
| *PP2C8* | F | ACAGTTTGGGAGCGAGAAGA | VIT_02s0025g01390 |
|  | R | CATTCCCTCCGAGACTCTCC |  |
| *PP2C37* | F | AGCCTTCTGAGACACGAACA | VIT_13s0019g02200 |
|  | R | CTTTCTTCCCGTCCTCCAGT |  |
| *PP2C51* | F | ATGTGGAAGACTGCTCGACA | VIT_16s0050g02680 |
|  | R | TACGACGGAGGCTGAAAACT |  |
| *PYL4* | F | CCCTCCAAAATCCTCGCTTG | VIT_08s0058g00470 |
|  | R | CCGCTTCTGAACTTGCTTGT |  |
| *SAPK2* | F | TCCTGCTAACACCAACCCAT | VIT_07s0191g00070 |
|  | R | ATCCTCGCTGAATCTACCGG |  |
| *BZR1* | F | CAGAAACCAAGCCCACCATC | VIT_18s0001g12020 |
|  | R | TGGGTTGGTAGGAAGCGAAT |  |
| *CYCD3-1* | F | GCAGTAAACCAAACGCTCCA | VIT_18s0001g09920 |
|  | R | CTAATGGGTTGCTTTCGGGG |  |
| *CYCD3-2* | F | TGTGAGGAGGAGAGATGGGA | VIT_03s0180g00040 |
|  | R | GCTTCCTCCTGCAAACACTC |  |
| *CYCD3-3* | F | GGGTTTTCTGCTCTGACTGC | VIT_07s0129g01100 |
|  | R | TGCAGGTCCAAGAGAAGAGG |  |
| *STPK* | F | CCTTCGGCTACTGTCTTTGC | VIT_02s0012g01140 |
|  | R | CCATCGTTGAGCATGTCCTG |  |
| *XTH22* | F | TACCCAAAGAACCAGCCCAT | VIT_11s0052g01180 |
|  | R | CCAGAAGACCAGATGCAAGC |  |
| *XTH23* | F | GTCCCATACCCGAAGAACCA | VIT_11s0052g01200 |
|  | R | CCAGAGGACCACAAACAAGC |  |
| *FSD2* | F | GTCGCCGTTGATGTACCTTC | VIT_16s0013g00260 |
|  | R | TGCGAGATGATTTCCACCCT |  |
| *FSD3* | F | GTTGCTGAGAAAAGTGGGCA | VIT_10s0042g00100 |
|  | R | TACAACCACTCGACTCACCC |  |
| *GSH-Px* | F | GTTGCTGAGAAAAGTGGGCA | VIT_05s0102g00120 |
|  | R | TACAACCACTCGACTCACCC |  |
| *PAO* | F | AGAGCAAACCCACGAACCTA | VIT_02s0025g04560 |
|  | R | CACATTCCTCAGCAATCCGG |  |
| *PAOX* | F | CCTTCCACACCCCTTGAGAT | VIT_01s0127g00800 |
|  | R | ATCCTCTCCGAAGTTGGCAA |  |
| *POD3* | F | GACACTGACCCTTCCATGGA | VIT_12s0059g02420 |
|  | R | GAAGAGCTGCATCGGATTGG |  |
| *POD29* | F | GTTTGGGGTTGAAGGCAGAG | VIT_05s0077g00880 |
|  | R | TCAAGAAAGCTGGAGGGGAG |  |
